# Low-Pressure Hydrocephalus After Traumatic Brain Injury: A Case Series and Scoping Review

**DOI:** 10.3390/jcm15187099

**Published:** 2026-09-13

**Authors:** Henry T. Beckett, Jorge Robles Solivan, Laura B. Ngwenya

**Affiliations:** 1 College of Medicine, University of Cincinnati, 3230 Eden Avenue, Cincinnati, OH 45267-0555, USA; becketht@mail.uc.edu (H.T.B.); roblesji@mail.uc.edu (J.R.S.); 2 Department of Neurosurgery, University of Cincinnati, 231 Albert Sabin Way, Cincinnati, OH 45267-0515, USA; 3 Department of Neurology & Rehabilitation Medicine, University of Cincinnati, Stetson Building Suite 2300, 260 Stetson Street, Cincinnati, OH 45267-0525, USA

**Keywords:** low-pressure hydrocephalus, hydrocephalus, traumatic brain injury, neurosurgery, scoping review

## Abstract

**Background/Objectives:** Low-pressure hydrocephalus (LPH) is an underappreciated pathology in which patients present with ventriculomegaly and intracranial pressures (ICPs) below normal, making traditional pressure-driven cerebrospinal fluid diversion treatment strategies challenging. LPH has been observed in patients following traumatic brain injury (TBI), often within the neurocritical care unit (NCCU). However, its presentation and associated patient characteristics remain unclear. Thus, we sought to compile patients from our institution and the literature to characterize LPH after TBI. **Methods:** Eleven patients were identified from our institutional chart review. Additionally, we performed a scoping review using PRISMA guidelines, which yielded eight publications, resulting in a total of 46 patients with LPH after TBI. **Results:** The median age was 34 years (IQR 23 to 50.75), with 86.96% male. When used, ventriculoperitoneal shunt (VPS) valves were programmed to a median pressure setting of 45 mmH_2_O (IQR 22 to 45). The median Glascow Outcome Scale-Extended (GOSE) score at 2–6 months post-shunt was 3 (IQR 2.25 to 4), indicating lower-severe disability and a 16.28% mortality. **Conclusions:** To our knowledge, this is the largest curation of TBI patients with LPH to date. From this pooled cohort, we observed that patients affected by LPH after TBI are often young to middle-aged and undergo multiple neurosurgical procedures prior to LPH diagnosis. Outcomes were poor with significant long-term disability and mortality. Clinicians should consider LPH diagnosis in TBI patients with hydrocephalus, especially if they have undergone prior neurosurgical procedures. Additionally, providers should recognize that these patients may potentially require lower opening pressures or certain pharmacological interventions compared to those with traditional post-traumatic hydrocephalus. Improved recognition of LPH after TBI can advance the understanding of the underlying mechanisms and inform treatment strategies.

## 1. Introduction

Hydrocephalus is a classic neurosurgical pathology characterized by a disruption in the production, flow, and/or absorption dynamics of cerebrospinal fluid (CSF) within the cranial vault, leading to ventriculomegaly [1]. A distinct subtype, originally described by Pang & Altschuler in 1994 as low-pressure hydrocephalus (LPH), has been comparatively less examined [2]. In contrast to more well-known forms of hydrocephalus such as obstructive or normal pressure, LPH is generally characterized by the presence of ventriculomegaly on imaging (i.e., Evan’s ratio ≥ 0.3) and low CSF pressures, typically below 50 to 80 mmH_2_O (3.67 to 5.88 mmHg), with negative pressures also possible (“negative-pressure hydrocephalus”), and it is often recognized when patients are in the neurocritical care unit (NCCU) [2,3,4,5]. Additionally, clinical features of hydrocephalus (e.g., headache, altered mental status, nausea/vomiting) are often present. Though no single factor has been identified to underlie LPH development, it is likely that physical disruption of the cranial vault and subarachnoid space are risk factors, including intracranial hemorrhage, neoplasm, previous neurological surgery, or trauma [4]. The largest case series to date on LPH, consisting of 195 patients, found that 22% of the adult cases were attributed to trauma [4]. Other case reports also contain patients with traumatic brain injuries (TBIs) leading up to the development of LPH [3,6]. However, the relationship between TBI, LPH, and other risk factors or patient characteristics has not been well established.

Despite the striking nature of this condition, the literature on LPH is limited by relatively scarce case reporting. The literature regarding LPH is further limited within the context of TBI, as the CSF changes normally associated with post-traumatic hydrocephalus (PTH) are akin to those with traditional hydrocephalus, compounded by factors such as intraventricular hemorrhage and infection [7]. However, typical PTH differs from LPH as there is usually elevated intracranial pressure (ICP) resulting from CSF outflow obstruction, often caused by the accumulation of blood products in the sub-arachnoid space. LPH does not have increased ICP, and this lack of elevated intracranial pressure may also fail to raise concern in the setting of trauma, given that a chief clinical priority is to detect and treat elevated pressures in order to avoid secondary complications such as herniation.

The premise of low- or negative-pressure hydrocephalus is counterintuitive given that most classic hydrocephalus cases consist of elevated CSF pressures, resulting in ventricular expansion. However, multiple theories exist around the pathophysiology of LPH and how alterations in tissue compliance, CSF flow dynamics, and other factors may contribute to enlarged ventricles even at low pressures [8,9]. The most widely accepted theory postulated by Pang & Altschuler concerns alterations of the viscoelastic properties (i.e., compliance and elasticity) of the brain parenchyma in the setting of chronic stretching and expulsion of extracellular fluid [2]. As compliance increases within the brain parenchyma, elasticity falls and lower changes in pressure are required to compress brain tissue, resulting in ventriculomegaly despite the lack of increased intraventricular pressures [7,10,11,12]. In fact, one case study using magnetic resonance elastography (MRE) measured a 46% decrease from normal brain stiffness in a patient with LPH [13].

Because of the unique low pressure, this makes the treatment and management of LPH challenging using conventional hydrocephalus treatment modalities. The lack of sufficient data surrounding LPH limits evidence to support best treatment practices. Additionally, it is unclear what risk factors may play a role in the development of LPH or in morbidity and mortality within this subset of patients with post-traumatic LPH. The objective of this case series and scoping review was to coalesce the available published cases to identify and describe patient and clinical characteristics of LPH following TBI, an underrecognized but clinically relevant phenomenon.

## 2. Methods

### 2.1. Protocol, Patient Selection and Eligibility Criteria

The case series was carried out via retrospective chart review with the approval of the Institutional Review Board and was exempt from informed consent. Patients from our institution were selected for inclusion via retrospective review of all shunt placements by a single neurologic surgeon at a large level 1 trauma center between 2016 and 2025. Only patients with confirmed LPH following traumatic brain injury were included, with LPH defined as the presence of ventriculomegaly on neuroimaging (CT/MRI) with concomitant CSF pressures equal to or below 70 mmH_2_O.

### 2.2. Information Sources and Search Strategy

**Figure 1:**
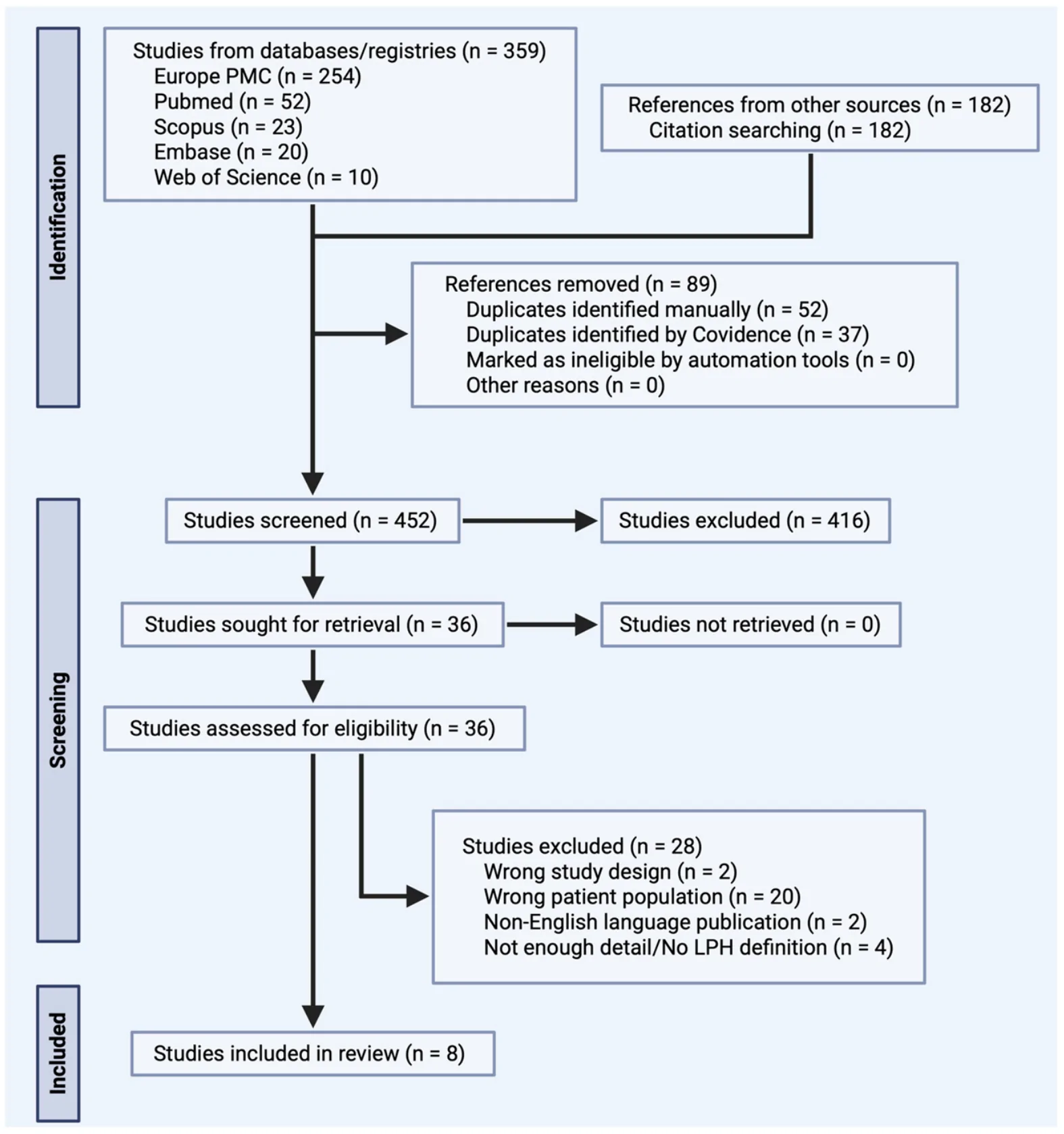
PRISMA review flow diagram.

The scoping review portion of this investigation was conducted using the Preferred Reporting Items for Systematic reviews and Meta-Analyses (PRISMA) extension for Scoping Reviews guidelines, following the PRISMA-ScR checklist (found in Supplementary Materials) with the assistance of Covidence software (https://www.covidence.org/, accessed on 9 September 2026) for study screening (Figure 1) [14,15]. Studies were included that discussed human subjects with confirmed LPH following traumatic brain injury, as previously defined. Additionally, included studies had to describe clinical features including treatment strategies (e.g., types of surgical interventions/shunt, external ventricular drain [EVD], etc.), CSF pressure measurements, and symptom manifestations. Given the relative paucity of data available, all age groups were included, and no publication date restrictions were imposed. During the search, no filters were imposed to restrict source/literature type in order to maximize the sensitivity of the search given the rarity of LPH following TBI. During screening, the objective was to identify sources of information with enough clinical data as outlined above to provide substantial enough data for descriptive analysis (e.g., abstracts, posters, case reports, case series, observational studies (cohort, cross-sectional, or retrospective studies), clinical trials, and experimental studies).

The following five databases were searched on 18 April 2025 for the primary analysis: PubMed, Embase, Scopus, Web of Science, and Europe PMC. An updated search was performed just prior to manuscript acceptance, on 8 August 2026. This search utilized identical search terms and strategy as outlined above and in the Supplemental Materials. Below is the search term used for the PubMed search. The search terms were modified as needed for database search formatting. For a complete list of the remaining individual database search terms, please see the provided Supplementary Document. Additionally, both forward and backward citation tracking was performed on the 8 articles that were selected from the literature search.

***PubMed***

*(“low pressure hydrocephalus” OR “LPH” OR “negative pressure hydrocephalus” OR “reversed gradient hydrocephalus” OR “ventricular overdrainage” OR “ventriculomegaly with low pressure” OR “paradoxical ventriculomegaly”)*

*AND*

*(“traumatic brain injury” OR “TBI” OR “brain trauma” OR “head trauma” OR “head injury” OR “brain injury” OR “post-traumatic hydrocephalus” OR “trauma-related hydrocephalus”)*

*NOT*

*(“normal pressure hydrocephalus” OR “NPH”)*

 

*With MeSH Terms:*

*(“low pressure hydrocephalus” OR “negative pressure hydrocephalus” OR “reversed gradient hydrocephalus” OR “ventricular overdrainage”)*

*AND*

*(“Brain Injuries” [MeSH Terms] OR “brain injury” OR “traumatic brain injury” OR “TBI” OR “head trauma” OR “post-traumatic hydrocephalus”)*

*NOT*

*(“normal pressure hydrocephalus” OR “NPH”)*

 

***Filters***

Date range: no restriction

Text availability: abstract, free full text, full text

Article types: all

### 2.3. Selection of Sources of Evidence

Two independent reviewers then screened titles and abstracts of articles returned from the search within Covidence, classifying each study for inclusion, maybe include, or exclusion. Conflicts were resolved via discussion and secondary review by both reviewers. Following the initial 2025 search, this resulted in 23 studies for full text review. The same two reviewers performed full text reviews of the 23 studies and resolved conflicts via discussion, leaving 6 articles for inclusion. Following the updated search, screening was performed identically to the process described during the initial search and screening (see above) on all new search results, following de-duplication. After this process and the performance of forward and backward citation tracking of the 6 articles that met the inclusion criteria during the initial search, an additional 13 sources were identified for full text review, bringing the total to 36 articles for full text review. From these, two additional sources met the criteria for inclusion, bringing the total to 8 included articles. No further sources were identified during forward and backward citation tracking of the two articles included following the updated search.

Studies were excluded if they included low-pressure hydrocephalus secondary to etiologies other than trauma (e.g., neoplasm, spontaneous intracranial hemorrhage, primary infection), were published in a language other than English, had incomplete case description, did not include confirmatory testing for LPH (i.e., ICP/CSF pressure readings, head imaging), or were not accessible for full text review. Additionally, studies on other forms of hydrocephalus, such as obstructive, normal pressure, and congenital, were excluded. Study types that were not allowed for inclusion in the final analysis were animal studies, editorials, commentaries, opinion pieces without clinical data or treatment discussion, expert opinions, or letters that do not provide primary or analyzable data. Ultimately, of the 36 studies that underwent full text review, 20 were excluded for wrong patient population (e.g., LPH outside of TBI), 4 were excluded for insufficient detail or no LPH definition (i.e., no ICP reported), 2 were excluded for incorrect study design (i.e., case series or review papers without available individual patient data for extraction), and 2 were excluded for non-English language publication.

### 2.4. Data Charting Process

Following screening, two reviewers independently and systematically extracted data from each of the 8 identified articles. The extracted data from each reviewer was then compared together for resolution of conflicts via discussion by the reviewers and added to a standardized spreadsheet. Not all data was uniformly available from each study, as reflected by the variable sample sizes for different variables seen in Table 1. Extracted variables included: age, sex, Glascow Coma Score (GCS) prior to treatment, time from TBI to LPH, time from LPH to definitive (therapeutic) shunt placement, total number of neurologic procedures (including EVDs), number of neurologic procedures prior to LPH (including EVDs), types of neurologic surgeries, shunt and valve type (brand), initial shunt valve settings, intracranial pressure measurements, Extended Glascow Outcome Score (GOSE; a clinical scale of post-TBI functional recovery), presence of central nervous system (CNS) infections, complications, and outcomes.

Data simplification and standardization were performed for all measurements. Pressure measurements for ICP and intracranial shunt valve settings were converted to mmH_2_O for standardization across all studies. Valve settings were converted to their corresponding pressure ranges (according to manufacturer reports) for statistical analysis. For valve settings that corresponded to a range of pressures, the average of the range was used for statistical calculations. Additionally, given that individual quantitative EVD pressure readings were not available for all patients, this was simplified to reflect either low (0 to 70 mmH_2_O) or negative (<0 mmH_2_O). Finally, again due to limited reporting of clinical details across studies, CNS infection was simplified into a binary (present or not) variable, rather than reporting of specific types of infection (hardware infection, subdural empyema, intraparenchymal abscess, etc.).

### 2.5. Synthesis of Results and Statistical Analysis

Data from the current series and collected studies were synthesized into a standardized table (Table S1). Data for each variable were assessed for normality. Descriptive statistics including the mean and standard deviation (SD) are reported for all normally distributed quantitative variables (Table 1). For non-normally distributed data, the median was calculated and reported with the interquartile range (IQR). Not all data were available across all studies, and missing data were handled using available-case analysis. Thus, patients who were missing data for a particular variable were removed from the analysis group for that variable but included for analysis of all variables for which they had available data.

## 3. Results

Our retrospective review identified 11 patients for inclusion from our institution. Of these, 9 (81.82%) were male and 2 were female. Their ages ranged from 16 to 34 with a median of 21 (IQR 19 to 24.5). The mechanisms of injury included motor vehicle crashes, pedestrians struck by motor vehicles, gunshot wounds, and head strikes. Initial GCSs upon presentation were uniformly in the severe category (between 3 and 8) with a median of 3 (IQR 3 to 5). The median time from TBI to LPH development was 41 days (IQR 27 to 67.5), and the median time from LPH development to shunt placement was 15 days (IQR 7 to 21). A total of 8 of 11 patients underwent hemicraniectomy during their treatment for TBI, with a median of 3 total neurosurgical procedures (including EVD placements; IQR 2 to 6) during their treatment course (both before and after LPH development). Nine patients received Sophysa SPV 140 low-pressure valves with their ventriculoperitoneal shunts (VPS) and two received Medtronic Strata NSC valves. One patient died within 6 months with a median GOSE score of 3 (IQR 3 to 4). Four of eleven patients developed subdural hygromas, and five of eleven had CNS infections during their treatment course. One patient had recorded ICPs in the negative range (−14 mmH_2_O), though four patients had a lowest recorded ICP of zero, with a median of 2 mmH_2_O (IQR 0 to 21).

The literature search and citation tracking returned 541 articles total. Thirty-seven studies were automatically identified by Covidence as duplicates, and 52 were identified manually during screening and removed, leaving 452 unique studies. Title and abstract screening yielded 36 studies for full text review, and, ultimately, 8 studies from the literature met the full criteria for inclusion in the scoping review (Figure 1). Those 8 publications included a total of 35 patients, resulting in 46 total patients for cumulative analysis when combined with the patients from our institution (Table 1). These studies were conducted in various countries, including the United States (Salam, 2025, Dalle Ore 2018, Michael 2017, Clarke 2006), Australia (Hunn, 2013), Canada (Houlden 2018), Spain (Godoy Hurtado 2023), and China (Wu 2019) (Table 2) [3,6,16,17,18,19,20,21]. The median age of patients from the literature (*n* = 35) was 40 years (IQR 29.5 to 56.5) with 31 males (88.57%). The median number of total neurosurgical procedures (including EVD placements) was 4 (IQR 2 to 6). 

When analysis was performed on all 46 patients, the ages ranged between 12 and 77 with a median age of 34 (IQR 23 to 50.75). Forty of the patients were male (86.96%). The median time from TBI to LPH development was 44 days (IQR 28 to 109.5; *n* = 17). The median time from LPH development to shunt placement was 101.5 days (IQR 23 to 211.25; *n* = 34). Seventeen patients had available data on neurosurgical procedures (including EVD placements) prior to LPH development with a median of 2 (IQR 1 to 4). Patients underwent a median of 3.5 total neurosurgical procedures, including EVD placements (IQR 2 to 6; n = 44). Forty-four of forty-six (95.65%) patients underwent either VPS (*n* = 42), ventriculoatrial shunt (VAS; *n* = 1), or ventriculopleural shunt (VPlS; *n* = 1) placement during their care for LPH with a median valve pressure setting of 45 mmH_2_O (IQR 22 to 45; *n* = 41, as 2 patients did not have shunts and 3 received valveless shunts). The median GOSE score at 2–6 months post-shunt was 3 (IQR 2.25 to 4), indicating lower-severe disability. There was a 16.28% mortality rate over the same 2–6-month post-shunt period.

## 4. Discussion

LPH following TBI is an underrecognized pathology involving ventriculomegaly with ICPs less than 70 mmH_2_O and may prove challenging for clinicians in diagnosis and management. This review was intended to compile the available cases of LPH after TBI from the literature alongside our own to identify clinical features, management strategies, and outcomes. This search resulted in 46 total cases and, to our knowledge, is the largest collection of LPH post-TBI cases to date. It is worth noting that the available literature was heterogeneous with respect to study design and the number of patients contributed from individual publications. The pooled cohort consisted of both case series and isolated case reports, which can result in unequal representation across publications. Given the limited literature on this condition, combining our institutional cases with published cases allows us to increase the sample size available for descriptive analysis. However, this does not omit differences in subject selection, treatment practices, or reporting practices between studies. Accordingly, the combined cohort should be interpreted as a descriptive compilation of post-traumatic LPH patients rather than a homogeneous cohort where population-level characteristics can be estimated. 

Key observations from affected patients include young to middle age at diagnosis, male predominance, and delayed symptom onset relative to TBI (weeks to months), with patients having undergone multiple neurosurgical procedures both before and after LPH diagnosis. Finally, observed treatment strategies relied primarily on low-pressure valve VPS systems with poor outcomes and high rates of functional neurologic disability and mortality, though without controls, it is difficult to assess whether outcomes are secondary to LPH or more related to the severity of TBI.

### 4.1. Clinical Characteristics and Potential Associated Factors

According to these data, it appears that patients affected by LPH after TBI were often young to middle-aged and underwent multiple neurosurgical procedures prior to LPH diagnosis. Interestingly, among other variables, older age is a considerable risk factor for the development of normal or elevated pressure PTH [22]. Thus, it is also plausible that age may represent a variable for what type of hydrocephalus patients are at risk of developing in the post-TBI period. However, given our relatively small sample size and the lack of a control group, we are unable to definitively name this as a risk factor for LPH following TBI.

When considering other factors associated with TBI, increased ICP secondary to cerebral edema, inflammation, and intracranial hemorrhage is common and was present in all eleven LPH patients from our own institution. The etiology of compliance and elasticity derangements underlying the development of LPH is yet to be determined. However, it is possible that alteration of the water content within the extracellular space is a contributing factor, as is seen with increased cerebral edema, and inflammation. If these processes are long standing, resulting in persistently elevated ICPs, it is reasonable to think that this may contribute to changes in the brain tissue water content and turgor, which could potentially predispose to the development of LPH.

In terms of the association between intracranial hemorrhage and LPH development, unfortunately, details regarding the presence and type of intracranial hemorrhage from the literature cases were limited. However, all 11 patients from our cohort had intracranial hemorrhage, with 10 of 11 having subarachnoid hemorrhage (SAH) +/- other types of hemorrhage, and at least 1 additional patient from the literature also suffered from multiple types of intracranial hemorrhage [16]. This is interesting, as it has been suggested that changes in brain compliance and elasticity may be explained by disrupted cerebral aquaporin-4 (AQP4) channels [11]. AQP4 is one of the primary mediators of water regulation within the brain/blood–brain barrier and is involved in the glymphatic drainage system, which is thought to be important for maintaining normal brain viscoelasticity [23]. SAH and the subsequent hematologic breakdown products may “clog” or otherwise disrupt AQP4 function within the brain, preventing the proper movement of water across the blood–brain barrier and between the brain parenchyma and subarachnoid space [24]. This is akin to the theory underlying normal-pressure hydrocephalus (NPH) pathophysiology, which involves damage to the arachnoid villi by SAH or meningitis [25]. In fact, similar to LPH, NPH has also been shown to demonstrate alterations in brain compliance and elasticity using MRE and may arise from similar pathophysiologic processes affecting brain turgor, albeit via different etiologies [26]. Furthermore, others have found acute hydrocephalus, intraventricular hemorrhage, and the Hunt and Hess grade to be predictive of chronic hydrocephalus and shunt dependency in alternate brain pathologies such as SAH [27,28]. While interesting and plausible, these findings and their relation to the underlying pathophysiology of LPH have yet to be appropriately modeled and confirmed by supporting basic science studies. Thus, they should be interpreted with healthy skepticism and represent areas of potential exploration moving forward.

Additionally, other TBI sequelae such as diffuse axonal injury (DAI) have been implicated as key contributors to progressive disability and mortality in the long term [29]. Similarly to LPH, the pathophysiology of DAI has not been entirely elucidated, yet its proposed mechanisms share considerable overlap with LPH, particularly regarding glymphatic and neurovascular dysregulation. In the setting of DAI, changes in AQP4 have been similarly implicated in glymphatic dysfunction and the diminished waste clearing capacity of the brain parenchyma following TBI [30]. These changes may provide a mechanistic link between DAI and disturbances in fluid homeostasis that potentially contribute to the development or persistence of post-traumatic hydrocephalus.

TBI patients also often have disruption to the cranial vault from skull fracture or surgical interventions, and of the 17 patients for whom detailed data were available on clinical course prior to LPH, all 17 had at least 1 neurosurgical operation or EVD placement prior to LPH. However, it is difficult to discern whether the procedures themselves pre-disposed patients to LPH or whether the pathophysiology underlying LPH was already established and they would have gone on to develop LPH regardless of any procedures. Additionally, we observed that the development of LPH occurred over weeks to months following TBI. It is likely that during this time, deleterious effects of edema, inflammation, hemorrhage, and surgical interventions cause disruption of the previously discussed homeostatic processes that maintain normal brain turgor. This is consistent with the high severity of TBI seen among patients who develop LPH, as they are more likely to suffer from contributory intracranial processes (e.g., edema, hemorrhage, etc.) and to require surgical interventions [31,32,33].

Finally, a large proportion of patients from our institutional cohort underwent permanent CSF diversion such as ventriculoperitoneal (VP) shunt, which may be related, in part, to the manner in which our institutional cohort of LPH patients was identified. Thus, the observed association between LPH and CSF diversion should be interpreted in the context of treatment workflow rather than as a proxy of prevalence in the broader patient population. However, in contrast to our institutional cohort, the identification of cases from the literature was not tied to shunt placement, and among those patients, 33 of 35 were treated with permanent CSF diversion shunt devices. Therefore, the frequency of CSF diversion within our identified cohort may still reflect an important feature of LPH, particularly if LPH is readily recognized in patients undergoing invasive monitoring or treatment.

### 4.2. Complications

#### 4.2.1. Infection

Infection rates in LPH were of particular interest given the possibility that infection could contribute to or result from LPH. The data available showed an overall infection rate of 43% throughout the clinical course, though data from the largest set of patients (Wu et al., 2019) did not specify if infection occurred before or after LPH diagnosis [6]. Thus, based on these data, it is not possible to establish a relationship beyond simple association between infection and patients with LPH. However, Wu et al., 2019 proposed an interesting hypothesis of LPH causing stagnant or even retrograde flow (in cases with negative pressure) of CSF within VPS systems as a potential contributor to infection risk [6]. Among our cohort, 6 of the 10 negative-pressure patients developed CNS infection compared to 13 of 36 among low-pressure patients. Though an interesting hypothesis, small samples warrant caution in interpretation of this trend. Among the patients from other studies with infections, three suffered infections prior to LPH development and four presented either with infection and LPH together or developed infection after LPH was already present. It is possible that infection may contribute to the development of LPH for similar reasons as how other causes of inflammation may alter the tissue composition and CSF flow dynamics. Furthermore, the proposed mechanism of infection from stagnant or retrograde CSF flow is possibly compounded by the inherent infection risk that accompanies VPS placement, which has been reported to range from 4–17%, as nearly all patients in the present work were treated with such systems and underwent multiple procedures [34].

#### 4.2.2. Parafalcine Hygroma

Four of the eleven patients from our institution developed subdural parafalcine hygromas prior to shunt placement. All four patients previously underwent hemicraniectomy. However, of the seven patients who did not develop hygromas, four of them also underwent hemicraniectomy. Interestingly, subdural hygromas have been associated with the development of hydrocephalus following decompressive hemicraniectomy in elevated pressure PTH [35]. Parallel to LPH, altered brain compliance has been proposed as a potential driver of this meningeal process, although the exact pathophysiology is not well established. This serves to highlight that subdural hygromas, although not necessary for diagnosis, are not exclusive to elevated pressure PTH, and LPH should not be excluded from the differential diagnosis when they are present.

### 4.3. Treatment Strategies and Challenges

Akin to LPH, normal or elevated pressure PTH has been thought to arise from various mechanisms. However, there is more consensus on the cause of normal and elevated pressures being a disruption of CSF absorption and/or CSF dynamics. It is this pressure differential that has historically informed patient management and served as a predictor of clinical improvement following CSF diversion. Traditional valvular shunt settings in pressure-driven CSF diversion techniques employed to treat normal- or high-pressure hydrocephalus did not seem to be effective in treating LPH. This is logical when considering these techniques’ reliance upon pressure gradients, which are absent or reversed in LPH. Patients often had continued or worsened clinical symptoms of hydrocephalus when treated with conventional valves and only showed signs of improvement when valves were programmed to low pressures (median of 45 mmH_2_O). Among our pooled cohort, shunt valves with very-low-pressure settings and those lacking anti-siphon mechanisms permitted clinical improvement in patients with LPH, which may suggest that some patients with LPH potentially require lower opening pressures compared to those with traditional PTH. Furthermore, valveless shunt systems permitted clinical improvement in most patients, though some patients remained symptomatic following the use of this hardware [16]. It is worth noting that these observed treatment patterns were largely provider dependent and are insufficient to establish one approach as superior over another, nor do they establish low-pressure systems as a treatment requirement. Additionally, the effectiveness of other surgical techniques such as endoscopic third ventriculostomy (ETV) specifically for the management of post-traumatic LPH is debatable, as one patient from our pooled cohort underwent ETV without improvement, though this intervention has shown some success in the broader LPH literature [4,16,36,37]. 

The employment of adjustable valve systems provides the ability to modify the pressure at which CSF is allowed to drain. This is also in keeping with consensus recommendations from a European expert panel in 2024, which said that programmable shunt valves are recommended over fixed pressure setting valves in post-traumatic hydrocephalus given this condition’s more dynamic course and need for adaptation of the system to individual patient requirements [38]. Additionally, they deemed that in patients who underwent decompressive hemicraniectomy, cranioplasty should be performed prior to deciding whether to place permanent CSF diversion devices for the treatment of ventriculomegaly, though concomitant placement of temporary external shunts during cranioplasty is still debated [38]. Because patients with post-traumatic LPH did not respond as well to traditional “default” valve settings, it is not surprising that patients underwent multiple trials at different valve settings prior to reaching an appropriately low setting to achieve clinical improvement or slow deterioration. This approach may be problematic, as it has been shown that in the absence of trauma, patients with normal-pressure hydrocephalus benefit more from sequential minor adjustments or a single large adjustment, as opposed to numerous drastic changes over a short period [39]. Thus, the effect of aggressive valve manipulations in post-traumatic LPH has yet to be elucidated, although previous studies on hydrocephalus in general may suggest that this does not serve to improve patient outcomes. This represents an area that would benefit from further investigation into the optimal titration of valve settings and pressures to improve clinical outcomes in LPH.

Beyond the use of hardware, pharmacologic agents such as theophylline have also shown some success in treating acute intracranial hypotension without the need for CSF diversion, though this patient experienced severe adverse effects including focal seizures that progressed to status epilepticus [16,40]. However, following the resolution of seizures, their LPH resolved, with ICP normalizing and a decrease in ventriculomegaly. Based on this, theophylline may be a useful tool for certain patients with PTH refractory to CSF diversion or when such procedures are contraindicated or multiple attempts have failed.

Historically, normal-pressure PTH has posed similar management challenges to LPH, particularly in diagnosis and gauging clinical responsiveness to CSF diversion. Specifically, authors have highlighted that diagnosing normal-pressure hydrocephalus in the setting of trauma is difficult due to patients often not being sufficiently arousable to display symptoms of hydrocephalus [41]. This is important to note, as obtunded patients with diminished intracranial pressures may also be diagnosed later, if at all, leading to a prolonged hydrocephalic state and sub-optimal clinical outcomes. Additionally, there is great focus on monitoring for increased ICP in TBI patients, especially within the NCCU. Thus, low pressures may be falsely reassuring against adverse intracranial processes. As LPH is underrecognized, it is likely that many patients with LPH are undertreated or their VPS systems are incorrectly managed given the limited provider awareness of the appropriate management modifications to traditional paradigms for this less widely known pathology. Thus, providers should consider LPH in TBI patients who have poor neurologic recovery alongside ventriculomegaly and low ICPs, especially when working in the NCCU.

### 4.4. Outcomes

Unfortunately, the observed outcomes among our cohort of LPH patients were poor, with the median GOSE at 2–6 months after TBI demonstrating Lower to Upper Severe Disability (GOSE 3–4). It is unclear whether this low level of recovery is directly due to the effects of LPH itself or are more reflective of the severity of brain injury. However, it is in keeping with the data from moderate and severe TBI at 6 months, which shows GOSE scores of less than 4 in around 30% of patients with moderate TBI and around 50% of patients with severe TBI at 6 months post-injury follow-up [42]. This could be further assessed in future retrospective studies comparing outcomes of patients who go on to develop LPH versus non-LPH controls who presented with similar injury mechanisms and initial clinical statuses. However, in reports of LPH outside of TBI alone, it does appear that treating LPH and resolving ventriculomegaly improves symptoms (e.g., altered level of consciousness, headache, nausea/vomiting, cranial nerve palsies) [2,4]. These poor outcomes are likely multifactorial in etiology, arising from TBI severity, delayed recognition of LPH, and treatment challenges. Mortality was slightly higher in our cohort compared to the largest systematic review of LPH patients by Keough, 2020 to date (16% vs. 11%) [4]. This is likely secondary to TBI being the primary insult in the present study compared to the mostly non-traumatic etiologies in the other study, as well as differences in sample sizes.

### 4.5. Limitations

The limitations of this study include a small sample size. Although this study utilized PRISMA guidelines for the review portion, only five databases were searched with the final inclusion of only eight articles. This likely results from both the under-recognition of LPH by clinicians and thus underreporting of cases in the literature, in addition to potentially missed cases by our search strategy. This is within the context of LPH being an entity that is not widely taught in standard medical education and thus does not possess standardized nomenclature. Our search terms sought to mitigate this by including a variety of terms that may be used to describe LPH.

This review was restricted to English-language studies, introducing the potential for language bias. However, only two potentially eligible non-English publication were excluded, suggesting that the effect of this criterion on the overall observed findings was likely limited. Due to incomplete reporting of individual variables across studies, an available-case analysis was used. As a result, sample sizes varied across analyses, and missing data may be a result of selective reporting practices commonly seen in case reports and case series. As such, variables with much smaller sample sizes should be interpreted cautiously. One should also consider that much of the literature on LPH pathophysiology is theoretical and without robust basic science data to support granular-level mechanisms. It is also difficult to assess the impact of LPH on outcomes given the severity of brain injury as a confounder. This would be better assessed in retrospective or prospective trials comparing LPH patients with varying levels of TBI severity. Lastly, the institutional cohort was derived from cases identified within the practice of a single surgeon, where the use of low-pressure VP shunt valves facilitated the recognition of LPH in patients with TBI. This ascertainment strategy may have augmented the cohort who underwent CSF diversion and therefore limits interpretation of the observed treatment frequency as representative of the broader LPH patient population.

## 5. Conclusions

LPH following TBI is a relatively uncommon, underrecognized pathology that, up to this point, has been poorly characterized. This study sought to synthesize the available data from the literature and our own patients to describe the characteristics of LPH and its management. Though it is unclear from these data what the contribution of LPH versus TBI severity is to long-term patient outcomes, clinicians should consider LPH diagnosis in TBI patients with hydrocephalus, especially if they have severe TBIs, have undergone prior neurosurgical procedures, and have persistent neurologic deficits with ventriculomegaly alongside low ICPs. These findings are relevant to considering how TBI and its associated clinical course may contribute to changes in brain compliance/elasticity leading to LPH and provide potential avenues for experiments exploring the mechanistic pathophysiology moving forward. Additionally, it should be recognized that certain patients with LPH may benefit from VPS or other CSF diversion systems that permit low pressure drainage, and emerging pharmacotherapy options represent additional directions for therapeutic exploration moving forward. Increased data reporting will enable a better understanding of the underlying mechanisms, which is needed to improve treatment strategies and outcomes for patients with LPH following TBI.

## Figures and Tables

**Table 1:** Demographic and clinical features of pooled post-traumatic LPH patients.

Characteristics	*n* (%) or Mean (SD) or Median [IQR]
Sex (*n* = 46/46)	
Male	40 (86.96%)
Female	6 (13.04%)
Median Age (*n* = 46/46)	34 [23 to 50.75]
Median Number of NSGY Procedures Prior to LPH * (*n* = 17/46)	2 [1 to 4]
Median Number of Total NSGY Procedures * (*n* = 44/46)	3.5 [2 to 6]
Decompressive Hemicraniectomy (*n* = 17/46)	11 (64.71%)
Median Time from TBI to Presentation of LPH [days] (*n* = 17/46)	44 [28 to 109.5]
Median Time from LPH to Definitive Shunt [days] (*n* = 34/46)	101.5 [23 to 211.25]
Median Initial GCS on Presentation with TBI (*n* = 12/46)	3 [3 to 5]
Median GCS Prior to Shunt (*n* = 38/46)	8.5 [7 to 11]
Treated with VP/VA/VPl Shunt (*n* = 46/46) ^#^	44 (95.65%) ^##^
Intracranial Pressure (n = 46/46)	
Low Pressure [0 to 70 mmH_2_O]	36 (78.26%)
Negative Pressure [<0 mmH_2_O]	10 (21.74%)
Median Lowest Measured ICP [mmH_2_O] (*n* = 17/46)	0 [−14 to 14]
Median Shunt Valve Setting Pressure [mmH_2_O] (*n* = 41/46)	45 [22 to 45]
Median GOS-E [2 to 6 months post-shunt placement] (*n* = 38/46)	3 [2.25 to 4]
Death within first 6 months after shunt (*n* = 43/46)	7 (16.28%)
CNS Infections (*n* = 44/46)	19 (43.18%)

SD = standard deviation, IQR = interquartile range, LPH = low-pressure hydrocephalus, NSGY = neurosurgical, TBI = traumatic brain injury, GCS = Glasgow Coma Scale, VP = ventriculoperitoneal, VA = ventriculoatrial, VPl = ventriculopleural, ICP = intracranial pressure, GOS-E = Glasgow Outcome Scale Extended, CNS = central nervous system. * Including surgical operations and EVD placements; ^#^ 40 VPS and 1 VAS. Two patients who did not receive VP/VA shunt only received EVD placement prior to death. ^##^ One patient had refractory ventriculomegaly/LPH to multiple VP/VA/VPl shunts and was definitively treated with theophylline [16].

**Table 2:** Study characteristics.

Authors	Journal	Geographic Origin	Study Design	Number of Patients
Beckett, 2026	Current	United States	Case Series	11
Clarke, 2006 [17]	Journal of Neurosurgery	United States	Case Series	1 of 2 included *
Dalle Ore, 2018 [18]	Journal of Craniofacial Surgery	United States	Case Report	1
Houlden, 2018 [19]	Journal of Neurological Surgery Part B: Skull Base	Canada	Case Series	1 of 4 included *
Hunn, 2013 [21]	Clinical Neurology and Neurosurgery	Australia	Case Series	2 of 8 included *
Godoy Hurtado, 2023 [3]	Journal of Clinical Medicine	Spain	Case Series	1 of 6 included *
Michael, 2017 [20]	Neurology	United States	Case Report	1
Salam, 2025 [16]	Journal of Neurosurgery: Case Lessons	United States	Case Report	1
Wu, 2019 [6]	World Neurosurgery	China	Case Series	27 of 39 included *

* Patients not included did not meet inclusion criteria and often were not TBI related.

## Data Availability

All collected data used for analysis are available within the Supplementary Materials documents.

## Supplementary Materials

The following supporting information can be downloaded at: https://www.mdpi.com/article/10.3390/jcm15187099/s1, Table S1: Total data set of patients, Full search strategy for all databases, PRISMA-ScR checklist.

## Abbreviations

The following abbreviations are used in this manuscript:

AQP4	Aquaporin-4
CNS	Central nervous system
CSF	Cerebrospinal fluid
EVD	External ventricular drain
ICP	Intracranial pressure
IQR	Interquartile range
GCS	Glascow Coma Score
GOSE	Extended Glascow Outcome Score
LPH	Low-pressure hydrocephalus
MRE	Magnetic resonance elastography
NCCU	Neurocritical care unit
PRISMA	Preferred Reporting Items for Systematic reviews and Meta-Analyses
PTH	Post-traumatic hydrocephalus
SAH	Subarachnoid hemorrhage
SD	Standard deviation
TBI	Traumatic brain injury
VAS	Ventriculoatrial shunt
VPS	Ventriculoperitoneal shunt
VPlS	Ventriculopleural shunt
ETV	Endoscopic third ventriculostomy
