# Supplementary material for: Low-Pressure Hydrocephalus After Traumatic Brain Injury: A Case Series and Scoping Review
Source: J Clin Med. 2026 Sep 13;15(18):7099. doi: 10.3390/jcm15187099 (PMC13607946; doi:10.3390/jcm15187099)
Supplement: Supplementary file 1 [file jcm-15-07099-s001.zip › jcm-4499987-supplementary.pdf]

Preferred Reporting Items for Systematic reviews and Meta-Analyses extension for Scoping Reviews (PRISMA-ScR) Checklist

SECTION	ITEM	PRISMA-ScR CHECKLIST ITEM	REPORTED ON PAGE #
**TITLE**			
Title	1	Identify the report as a scoping review.	1
**ABSTRACT**			
Structured summary	2	Provide a structured summary that includes (as applicable): background, objectives, eligibility criteria, sources of evidence, charting methods, results, and conclusions that relate to the review questions and objectives.	1
**INTRODUCTION**			
Rationale	3	Describe the rationale for the review in the context of what is already known. Explain why the review questions/objectives lend themselves to a scoping review approach.	2, 3
Objectives	4	Provide an explicit statement of the questions and objectives being addressed with reference to their key elements (e.g., population or participants, concepts, and context) or other relevant key elements used to conceptualize the review questions and/or objectives.	3
**METHODS**			
Protocol and registration	5	Indicate whether a review protocol exists; state if and where it can be accessed (e.g., a Web address); and if available, provide registration information, including the registration number.	3
Eligibility criteria	6	Specify characteristics of the sources of evidence used as eligibility criteria (e.g., years considered, language, and publication status), and provide a rationale.	3
Information sources*	7	Describe all information sources in the search (e.g., databases with dates of coverage and contact with authors to identify additional sources), as well as the date the most recent search was executed.	3, 4
Search	8	Present the full electronic search strategy for at least 1 database, including any limits used, such that it could be repeated.	3, 4
Selection of sources of evidence†	9	State the process for selecting sources of evidence (i.e., screening and eligibility) included in the scoping review.	5
Data charting process‡	10	Describe the methods of charting data from the included sources of evidence (e.g., calibrated forms or forms that have been tested by the team before their use, and whether data charting was done independently or in duplicate) and any processes for obtaining and confirming data from investigators.	5,6
Data items	11	List and define all variables for which data were sought and any assumptions and simplifications made.	5, 6
Critical appraisal of individual sources of evidence§	12	If done, provide a rationale for conducting a critical appraisal of included sources of evidence; describe the methods used and how this information was used in any data synthesis (if appropriate).	n/a
Synthesis of results	13	Describe the methods of handling and summarizing the data that were charted.	6

Table S1. Total data set of patients

Age	Sex	Initial GCS	GCS Prior to Shunt	Time from TBI to LPH (Days)	Time from LPH to Definitive Shunt (days)	Total Number of NSGY Procedures**	Number of NSGY Procedures prior to LPH**	DHC	Shunt Valve	Valve Setting (pressure mmH2O)	EVD Pressure ***	Lowest Measured ICP (mmH2O)	GOS-E 2-6 Months Post-Shunt	Survival at 6 Months Post-Shunt	CNS Infection	Pre-Shunt PFH
32	M	5	6	91	54	9	4	Y	Sophysa SPV 140	1 (10)	Low	68	4	Y	Y	Y
21	F	5	15	32	15	2	2	N	Sophysa SPV 140	2 (40)	Low	0	4	Y	N	N
19	M	3	4	109	8	1	1	N	Sophysa SPV 140	1 (10)	Low	0	3	Y	N	N
16	M	3	4	29	5	2	4	Y	Sophysa SPV 140	1 (10)	Low	14	3	Y	N	Y
17*	M	6	7	25	2	2	2	Y	Sophysa SPV 140	(40)	Low	0	1	N	N	Y
23	M	3	6	41	12	6	3	Y	Sophysa SPV 140	(40)	Negative	-14	-	Y	Y	Y
21	M	3	9	41	26	2	2	Y	Sophysa SPV 140	3 (80)	Low	28	3	Y	Y	N
19	F	3	11	17	20	4	5	Y	Sophysa SPV 140	3 (80)	Low	68	-	Y	Y	N
26*	M	8	11	44	16	2	1	N	Sophysa SPV 140	2 (40)	Low	0	7	Y	Y	N
23	M	3	3	20	20	6	1	Y	Medtronic Strata NSC	0.5 (22)	Low	2	3	Y	N	N
34	M	3	9	111	6	6	4	Y	Medtronic Strata NSC	0.5 (22)	Low	2	-	Y	N	N
40	F	-	13	2190	-	14	7	N	Valveless VP Shunt	-	Low	0	3	Y	N	-
56	M	-	-	78	-	2	1	N	Medtronic Strata NSC	0.5 (22)	Negative	-	1	N	N	-
16	M	-	-	122	-	4	2	Y	Codman CERTAS VA	122	Negative	-	5	Y	Y	-

17	M	-	-	140	-	7	3	Y	Medtronic Strata II	0.5 (15-25)	Negative	-	1	N	N	-
30	F	-	3	15	-	4	1	N	Valveless VP Shunt	-	Low	50	8	Y	N	-
23	M	-	-	-	252	6	-	-	- (EVD)	-	Negative	-100	2	Y	Y	-
69	M	-	-	-	-	6	-	-	- (EVD)	-	Negative	-100	1	N	Y	-
29	M	-	7	-	185	4	-	-	CodMan Hakim	(30)	Negative	-50	3	Y	Y	-
34	M	-	11	-	62	2	-	-	Medtronic Strata II	0.5 (15-25)	Low	-	3	Y	N	-
37	M	-	8	-	101	7	-	-	Medtronic Strata II	0.5 (15-25)	Low	-	3	Y	N	-
36	M	-	9	-	209	5	-	-	Medtronic Strata II	0.5 (15-25)	Low	-	3	Y	Y	-
25	F	-	15	-	1134	2	-	-	Medtronic Strata II	0.5 (15-25)	Low	-	5	Y	Y	-
48	M	-	7	-	310	7	-	-	Medtronic Strata II	0.5 (15-25)	Low	-	2	Y	Y	-
42	F	-	14	-	108	2	-	-	Medtronic Strata II	0.5 (15-25)	Low	-	-	-	Y	-
39	M	-	8	-	-	7	-	-	Medtronic Strata II	1 (35-55)	Low	-	1	N	Y	-
31	M	-	7	-	212	10	-	-	Medtronic Strata II	1 (35-55)	Low	-	4	Y	Y	-
18	M	-	7	-	90	4	-	-	Medtronic Strata II	1 (35-55)	Low	-	3	Y	Y	-
45	M	-	12	-	116	2	-	-	Medtronic Strata II	1 (35-55)	Low	-	3	Y	N	-
59	M	-	9	-	730	3	-	-	Medtronic Strata II	1 (35-55)	Low	-	2	Y	N	-
51	M	-	8	-	494	2	-	-	Medtronic Strata II	1 (35-55)	Low	-	4	Y	Y	-
57	M	-	14	-	344	4	-	-	Medtronic Strata II	1 (35-55)	Low	-	3	Y	N	-
65	M	-	13	-	31	1	-	-	Medtronic Strata II	1 (35-55)	Low	-	3	Y	N	-
54	M	-	10	-	155	3	-	-	Medtronic Strata II	1 (35-55)	Low	-	4	Y	N	-
57	M	-	8	-	102	2	-	-	Medtronic Strata II	1 (35-55)	Low	-	4	Y	N	-
24	M	-	9	-	45	4	-	-	Medtronic Strata II	1 (35-55)	Low	-	5	Y	Y	-

67	M	-	5	-	-	2	-	-	Medtronic Strata II	1 (35-55)	Low	-	1	N	N	-
77	M	-	9	-	137	2	-	-	Medtronic Strata II	1 (35-55)	Low	-	4	Y	N	-
49	M	-	7	-	-	2	-	-	Medtronic Strata II	1 (35-55)	Low	-	1	N	N	-
26	M	-	7	-	140	2	-	-	Medtronic Strata II	1 (35-55)	Low	-	-	-	N	-
71	M	-	8	-	82	2	-	-	Medtronic Strata II	1 (35-55)	Low	-	3	Y	N	-
59	M	-	12	-	3880	8	-	-	Medtronic Strata II	1 (35-55)	Low	-	3	Y	N	-
50	M	-	12	-	556	3	-	-	Aesculap VP Shunt	(50)	Low	-	4	Y	N	-
30	M	6	-	90	-	12	3	Y	Codman CERTAS VA	-	Negative	-285	-	-	Y	-
12	M	-	-	-	-	-	-	-	Medtronic Strata II	1 (35-55)	Negative	-	-	Y	-	-
36	M	-	-	-	-	-	-	-	Medtronic Strata NSC	0.5 (22)	Negative	-	-	Y	-	-

LPH = Low Pressure Hydrocephalus, NSGY Procedures = Neurosurgical Procedures, DHC = Decompressive Hemicraniectomy, EVD = Extraventricular Drain, GCS = Glasgow Coma Scale, GOS-E = Glasgow Outcome Scale Extended, Y = Yes, N = No, "-" = Unknown/unavailable, PFH = Parafalcine Hygroma

*Confirmed CSF Leak
**Including EVDs
***"Low" = 0 to 70mmH2O

Search Strategy:

Pubmed

Search term:

("low pressure hydrocephalus" OR "LPH" OR "negative pressure hydrocephalus" OR "reversed gradient hydrocephalus" OR "ventricular overdrainage" OR "ventriculomegaly with low pressure" OR "paradoxical ventriculomegaly")

AND

("traumatic brain injury" OR "TBI" OR "brain trauma" OR "head trauma" OR "head injury" OR "brain injury" OR "post-traumatic hydrocephalus" OR "trauma-related hydrocephalus")

NOT

("normal pressure hydrocephalus" OR "NPH")

With MeSH Terms:

("low pressure hydrocephalus" OR "negative pressure hydrocephalus" OR "reversed gradient hydrocephalus" OR "ventricular overdrainage")

AND

("Brain Injuries"[MeSH Terms] OR "brain injury" OR "traumatic brain injury" OR "TBI" OR "head trauma" OR "post-traumatic hydrocephalus")

NOT

("normal pressure hydrocephalus" OR "NPH")

Filters:

Date range: no restriction

Text availability: abstract, free full text, full text

Article types: all

Embase

Search term:

('low pressure hydrocephalus' OR 'negative pressure hydrocephalus' OR 'reversed gradient hydrocephalus' OR 'ventricular overdrainage' OR 'ventriculomegaly with low pressure' OR 'paradoxical ventriculomegaly')

AND

('traumatic brain injury' OR 'TBI' OR 'brain trauma' OR 'head trauma' OR 'head injury' OR 'brain injury' OR 'post-traumatic hydrocephalus' OR 'trauma-related hydrocephalus')

NOT

('normal pressure hydrocephalus' OR 'NPH')

With Emtree Terms:

('low pressure hydrocephalus'/exp OR 'negative pressure hydrocephalus'/exp)

AND

('brain injury'/exp OR 'traumatic brain injury' OR 'TBI' OR 'post-traumatic hydrocephalus')

NOT

('normal pressure hydrocephalus' OR 'NPH')

Filters:

Date range: no restriction

Text availability: abstract, free full text, full text

Article types: all

Scopus

Search term:

(TITLE-ABS-KEY("low pressure hydrocephalus" OR "LPH" OR "negative pressure hydrocephalus" OR "reversed gradient hydrocephalus" OR "ventricular overdrainage" OR "ventriculomegaly with low pressure" OR "paradoxical ventriculomegaly")

AND

TITLE-ABS-KEY("traumatic brain injury" OR "TBI" OR "brain trauma" OR "head trauma" OR "head injury" OR "brain injury" OR "post-traumatic hydrocephalus" OR "trauma-related hydrocephalus"))

AND NOT

TITLE-ABS-KEY("normal pressure hydrocephalus" OR "NPH")

Filters:

Date range: no restriction

Text availability: abstract, free full text, full text

Article types: all

Web of Science

Search term:

TS=("low pressure hydrocephalus" OR "LPH" OR "negative pressure hydrocephalus" OR "reversed gradient hydrocephalus" OR "ventricular overdrainage" OR "ventriculomegaly with low pressure" OR "paradoxical ventriculomegaly")

AND

TS=("traumatic brain injury" OR "TBI" OR "brain trauma" OR "head trauma" OR "head injury" OR "brain injury" OR "post-traumatic hydrocephalus" OR "trauma-related hydrocephalus")

NOT

TS=("normal pressure hydrocephalus" OR "NPH")

Filters:

Date range: no restriction

Text availability: abstract, free full text, full text

Article types: all

Europe PMC

Search term:

("low pressure hydrocephalus" OR "LPH" OR "negative pressure hydrocephalus" OR "reversed gradient hydrocephalus" OR "ventricular overdrainage" OR "ventriculomegaly with low pressure" OR "paradoxical ventriculomegaly")

AND

("traumatic brain injury" OR "TBI" OR "brain trauma" OR "head trauma" OR "head injury" OR "brain injury" OR "post-traumatic hydrocephalus" OR "trauma-related hydrocephalus")

NOT

("normal pressure hydrocephalus" OR "NPH")

Filters:

Date range: no restriction

Text availability: abstract, free full text, full text

Article types: all
